# Development and evaluation of the efficacy of a web-based ‘social norms’-intervention for the prevention and reduction of substance use in a cluster-controlled trial conducted at eight German universities

**DOI:** 10.1186/s12889-016-2898-z

**Published:** 2016-03-11

**Authors:** Stefanie M. Helmer, Saskia Muellmann, Hajo Zeeb, Claudia R. Pischke

**Affiliations:** Leibniz Institute for Prevention Research and Epidemiology - BIPS, Bremen, Germany

## Abstract

**Background:**

Previous research suggests that perceptions of peer substance use are associated with personal use. Specifically, overestimating use in the peer group is predictive of higher rates of personal substance use. ‘Social norms’-interventions are based on the premise that changing these misperceived social norms regarding substance use by providing feedback on actual norms is associated with a reduction in personal substance use. Studies conducted in the U.S.A. suggest that ‘social norms’-feedback is an effective strategy for reducing substance use among university students. It is unknown whether the effects of a ‘social norms’-feedback on substance use can be replicated in a sample of German university students. The objective of this article is to describe the study design and aims of the ‘INternet-based Social norms-Intervention for the prevention of substance use among Students’ (INSIST)-study, a cluster-controlled trial examining the effects of a web-based ‘social norms’- intervention in students enrolled at four intervention universities with those enrolled at four delayed intervention control universities. The INSIST-study is funded by the German Federal Ministry of Health.

**Methods/Design:**

Eight universities in four regions in Germany will take part in the study, four serving as intervention and four as delayed intervention control universities (randomly selected within a geographic region). Six hundred students will be recruited at each university and will be asked to complete a web-based survey assessing personal and perceived substance use/attitudes towards substance use at baseline. These data will be used to develop the web-based ‘social norms’-feedback tailored to gender and university. Three months after the baseline survey, students at intervention universities will receive the intervention. Two months after the launch of the intervention, students of all eight universities will be asked to complete the follow-up questionnaires to assess changes in perceptions of/attitudes toward peer substance use and rates of personal substance use.

**Discussion:**

This study is the first German cluster-controlled trial investigating the influence of a web-based ‘social norms’-intervention on perceptions of/attitudes towards substance use and substance use behavior in a large university student sample. This study will provide new information on the efficacy of this intervention strategy in the German university context.

**Trial registration:**

DRKS00007635 at the ‘German Clinical Trials Register’ (17.12.2014).

## Background

Licit and illicit substance use (i.e., using alcohol, tobacco, cannabis and other illicit substances) is considered a key public health concern among university students. In regard to licit substance use, findings of previous studies suggest that university students tend to consume more alcohol than young adults of similar ages who are not studying [1, 2]. While daily drinking is comparatively low (4 % reported consumption of alcoholic beverages every day during the last three months), heavy drinking is common among German university students [3]. Keller and colleagues [4] estimated a 62 % three month heavy drinking prevalence among German university students. In another study, 16 % of German university students reported that they practiced heavy drinking at least once per week [3]. In regard to illicit substance use, one study reported a lifetime prevalence of 40 % for cannabis use in a sample of 3307 German university students [5]. Because university students are more likely to engage in unhealthy or risky substance use, such as heavy drinking, they are at a particular risk for substance use-related consequences, such as vandalism or sexual violence [6].

Results of a literature review summarizing findings of 65 articles examining the role of students’ characteristics in relation to alcohol use at European campuses indicate that alcohol is mainly consumed during social gatherings and that social motives for drinking prevail in this population [7]. Substance use is perceived to be a normal part of students’ life and personally engaging in substance use is perceived to be an adequate behavior to match the norm of peer behavior and therefore maintain conformity with peers [8]. However, international research suggests that university students tend to overestimate both the perceived quantity (*descriptive norm*) [9–11] and perceived acceptability (*injunctive norm*) [12] of alcohol and other substances used by their peers.

Inaccurate perceptions can cause the individual to increase their own consumption in an attempt to match their personal behavior to the peer norm. Overestimations of peer licit [9, 10, 13] and illicit substance use [14–16] and associations with increased personal substance use among university students have been demonstrated in several previous studies [9, 13, 16].

Based on this observation, so called ‘social norms’-interventions were developed which aim to change university students’ perceptions of attitudes towards substance use among their peers and of peer substance use, using feedback [17]. Typically, participants of ‘social norms’-interventions are exposed to feedback contrasting their perception of substance use and attitudes towards use among their peers with data on actual use and attitudes in their peer group. Data on perceived attitudes and use, as well as actual attitudes and use, are assessed prior to the development of the feedback. Findings of ‘social norms’-intervention studies suggest that providing feedback on actual peer consumption rates to students leads to a reduction of social pressure on the individual and may consequently reduce personal substance use [18, 19]. ‘Social norms’-interventions have been delivered employing a mass media marketing approach [20], have targeted individuals [21] and have been implemented in group counselling sessions [22]. In more recently developed interventions, ‘social norms’-feedback was delivered via the internet [18, 19]. One advantage of web-based interventions is that the messages can be personalized. Feedback on actual peer norms can be based on national norms [22], behavior and attitudes of university students at a person’s university [23] and can also be presented gender neutral [24] or stratified for same-gender peers [25].

To date, ‘social norms’-intervention research has largely been restricted to the North-American college system and little is known about how perceptions of peer use influence personal substance use in European students [8]. The ‘Social Norms Intervention for the prevention of Polydrug usE’ (SNIPE) study was the first multi-national study that aimed to develop an online ‘social norms’-intervention portal which was implemented in seven European countries [26]. In the present study, this intervention will be translated and adapted to the German university context. The main aim of the study is to investigate whether participation in this intervention leads to greater changes in misperceived social norms and a more pronounced reduction of licit and illicit substance use among German university students enrolled at four intervention universities compared to students enrolled at four delayed intervention control universities. This cluster-controlled trial is funded by the German Federal Ministry of Health.

## Methods

### Study aims

The primary objectives of the INSIST (‘INternet-based Social norms-Intervention for the prevention of substance use among Students’)-study are to evaluate (I) whether misperceptions of peer substance use are reduced as a consequence of participating in the intervention and (II) whether students participating in the intervention report lower rates of substance use at follow-up than those not participating in the intervention. Differences between the estimates (norms and use of substances) of students of the intervention and the control group are used as indicators to measure possible intervention effects.

Secondary objectives of the INSIST-study include:

(i) The detailed assessment of self-reported consumption rates of licit and illicit substances in a large sample of German university students;
(ii) the adaption of an internationally implemented ‘social norms’-intervention to the needs of German university students using qualitative results of focus group discussions with university students.

### Participants and procedures

Ethical approval was obtained from institutional review boards of all participating universities (Research and Ethics Committee of the Department of Life Sciences, HAW Hamburg; MHH Ethics Commission, Hannover Medical School; Ethics Commission of the University of Münster provided approval for the study site at the University of Bielefeld; Ethics Commission of the Heinrich Heine University Düsseldorf; Ethics Commission of the Martin Luther University Halle-Wittenberg; Ethics Commission of the Technical University Dresden; Medical Ethics Commission II of Mannheim University provided approval for the study site at Heidelberg University and Mannheim University). In addition, issues regarding data protection were monitored by the local data protection agency in the city state of Bremen. Eight universities in four regions will participate in the study (Hamburg University of Applied Sciences, Hannover Medical School, University of Bielefeld, Heinrich Heine University Düsseldorf, Martin Luther University Halle-Wittenberg, Technical University Dresden, Heidelberg University, and Mannheim University). In each region, one university will serve as the intervention site and one as the comparison site. Within a geographical area intervention and control universities will be determined by random selection. However, intervention and control universities in each of the four regions will be located in different cities. Social contacts between students of these two universities are expected to be minimal, as well as the likelihood of cross-contamination of intervention effects.

Students of all participating universities will be invited to participate in the INSIST-study. For the recruitment of students, an overall recruitment strategy across all universities will be employed. At each university, one local student will be employed by the study who will be in charge of recruitment at the university. We expect that these students will be familiar with structures and processes at their universities which may facilitate recruitment. Students will be recruited to the study via email, the universities’ websites, intranet or student e-learning platforms. Additional public recruitment channels will include local newspaper articles, local radio broadcasts, and student newsletters. Moreover, students will be personally invited to participate in the study in seminars by lecturers. Posters and flyers promoting the study will be posted at universities and Facebook will be used to publicize the study.

To participate in the INSIST-study, students will be asked to firstly register for the study via email. The email will contain a hyperlink to the survey website where students can enter their email-address and choose their respective university and gender (female, male, or other). This information is necessary to create the individualized university- and gender-specific’social norms’-feedback. Students will be informed on the study website that they can withdraw from the study at any time. If they register and choose to take part in the study, students will receive an email with information regarding the date the baseline survey is available. After the survey is launched, registered students will be informed via email that they can fill out the baseline questionnaire.

### Sample size

The sample size calculation is based on the prevalence for heavy drinking reported in a previous study conducted in Germany with 3307 students [5]. In total, we aim to reach 4800 students at baseline, 600 students at each participating university. We assume a 40 % loss to follow-up five months post-baseline. The sample size will allow an estimation of a difference in the rate of heavy drinking between the intervention and control sites (at follow-up), corresponding to an effect size of 0.2 at the level of p < .05 with 81 % power.

### Design of the baseline questionnaire

We will employ a web-based survey. The advantage of a web-based survey is that it is anonymous and that the questionnaire can be personalized to students’ gender and university. University students are a computer-literate population. A web-based survey is therefore a reliable survey tool [27]. Baseline and follow-up questionnaires will be identical for both, intervention and delayed intervention control universities. The questionnaires will include items assessing socio-demographic information, such as age, migration background, if applicable, length of stay in the respective country, whether a student came to study to the respective country, religion and importance of religion, place of residence, disposable weekly income, disposable weekly income spent on licit and illicit substances. Furthermore, information on subject and year of study will be assessed.

Students will be asked to answer questions regarding their personal and perceived substance use of peers and their personal attitude and their perceptions of the attitudes of their peers towards using the following substances: Alcoholic beverages, tobacco products, waterpipe, cannabis, non-prescribed medications to improve academic performance, non-prescribed sedatives or sleeping pills, synthetic cannabis, cocaine, ecstasy, other amphetamine-type stimulants, hallucinogens, and inhalants. Licit and illicit substances will be followed by a list of examples and, if applicable, trade or street names for each substance. Regarding alcohol use, the following variables will be assessed: Average and maximum number of alcoholic drinks consumed on a day that alcohol is consumed, number of occasions where students drink until they feel drunk. Along with the items on alcohol use, participants will be provided with a definition of an alcoholic drink as 0.33 liter beer, a small bottle of a ready to drink beverage (0.275 l), a small cocktail (0.2 l, containing 4 cl alcohol), a glass of wine/sparkling wine (0.125 l), and a shot of spirits (0.4 l). Furthermore, two types of polydrug use will be assessed (i.e., simultaneous use of alcohol and tobacco, alcohol and illicit substances, such as cannabis, ecstasy or cocaine).

Referring to this range of substances, students will be asked to report their personal substance use behaviors and attitudes towards substance use and the perceived gender-specific behaviors (*descriptive norm*) and attitudes (*injunctive norm*) of their peers.

Apart from these questions, an adapted version of the CORE Alcohol and Drug Survey Long Form [28] will be used to assess negative consequences of using alcohol or illicit substances (e.g., missing a class or another commitment, unprotected sex, engagement in violent acts). Finally, students will be asked how they heard about the survey (e.g., via email, information during a lecture, social media channel). This will contribute to an improvement in the selection of suitable recruitment channels in future surveys targeting university students. All questions will be referring to a time period of three months prior to assessment. After completing the questionnaire, students will receive the contact information of the researcher who coordinates the study and who can be contacted for project-related questions. Furthermore, they will be informed about local drug counselling services (at their university and available outside the university setting) that can be contacted if they require counselling regarding their personal substance use behavior.

### Use of a web-based social norms-approach

The implementation of a personalized web-based, gender-specific, normative intervention is the central part of the INSIST-study. We will develop a gender-specific normative feedback for the intervention group (i.e., students enrolled at the four intervention universities) that will be based on previously assessed baseline data regarding descriptive and injunctive norms related to substance use at the respective university. Students from the intervention universities who registered for the study will be invited by email to check their personalized normative feedback approximately two months after the baseline survey opens. Students will be informed that they can access their personalized feedback multiple times. The feedback website will consist of several different main pages that will be accessible via a navigation menu. Each main page will contain information about a different substance (i.e., alcohol, tobacco, cannabis). All main pages will be divided into a personalized feedback and a gender- and university-specific feedback. The personalized feedback will include the individual information regarding own substance use and the perception of use in the peer group (same gender, same university) reported by students. If students do not fill out the respective questions of the baseline questionnaire beforehand, they will be informed that an individual feedback cannot be displayed because of missing data. The gender- and university-specific feedback will visualize the perceived peer substance use (of the majority of students of the same gender, same university) that was estimated by the survey participant. This information will be contrasted with the actual substance use pattern of students of the same gender and same university that was assessed in the baseline questionnaire. These two comparisons will form the *descriptive norms* feedback. Furthermore, students will receive information about the *injunctive norms* of same-gender peers at their universities. This information will be displayed on the feedback website in corresponding information boxes.

### Follow-up survey and delayed intervention for participants at control universities

The follow-up survey will take place approximately five months post-baseline and the same items will be employed. The only difference will be that students will not be asked in the follow-up survey how they heard about the study but students at the intervention universities will be asked whether they remember the content of the normative feedback. To illustrate the question, an example from the normative feedback will be used. At the end of the study, students at the delayed intervention control universities will be provided access to the intervention.

### Qualitative research informing the design of the intervention

Focus group discussions with the target group will be held to gather suggestions regarding the suitability of the INSIST-questionnaire and the web-based gender-specific normative feedback. Specifically, German students will be asked questions regarding their opinions on the reference groups used in the questionnaire. In addition, students will be asked who they consider to be a “typical student” and whether they think that their peers can identify with this term. Further, they will be asked to provide their opinion regarding different categories for determining a ‘majority of students’ (e.g., >51 %, or a value between 0–10 or 0–100 of male or female peers at their university). Focus group discussions will be led by a researcher of the INSIST team using a previously developed focus group guide. Attendance of six to ten university students will be intended per focus group discussion. Participants will be recruited using the snowball-method. Researchers leading the focus groups will obtain an informed consent by focus group participants before starting the discussion.

### Analysis strategy

#### Quantitative analysis

Self-other discrepancies in perceptions of substance use and personal substance use behavior will be evaluated by analyzing whether students perceived the substance use of the majority of their peers to be lower/equal/higher than their personal substance use in bivariate analyses. Cross-sectional associations between perceptions of peer substance use and personal substance use at baseline will be analyzed using logistic regression. To evaluate intervention efficacy, substance use pre- and post-intervention among students at intervention universities will be compared to the use reported by students enrolled at delayed intervention control universities. The same approach will be taken for the analysis of changes in the descriptive and injunctive norms related to substance use for the different substances over time. Both analyses will involve bivariate tests and generalized linear models. In addition, structural equation models will be used to assess relationships between changes in norms and substance use behaviors, similar to previous studies investigating efficacy of ‘social norms’-interventions [28]. SAS statistical software [29] will be used to undertake quantitative analyses.

#### Qualitative analysis

Qualitative focus group discussions will be protocolled by two researchers and audio-recorded. The audio-record will be transcribed using the software f4. Protocols will be compared and complemented by listening to the audio record. Both deductive and inductive methods will be used for analyzing the data. Based on the interview guide an analysis matrix with different categories will be developed. The contents of the final protocols will be transferred to each category of the analysis matrix.

### Process evaluation and external advisory board

Researchers of an external institution will evaluate the recruitment and data collection process and will monitor intervention implementation. Process evaluation data will be summarized by the research team of the institute. Furthermore, an international advisory board, consisting of ‘social norms’ researchers will supervise the INSIST-study and will provide advice before and during the study. The INSIST research team will provide status updates on the project to the advisory board at least twice during the funding period.

### Expected results

Regarding the baseline survey, we expect to find that students perceive peer licit and illicit substance use to be higher than their personal use. We base this assumption on findings of previous studies conducted in North America (see above) and misperceptions found in European students in the SNIPE study [13, 16, 30]. In regard to intervention efficacy, we expect to detect effects of the intervention on perceptions of/attitudes towards peer substance use and personal substance use, i.e., more pronounced reductions in misperceptions of/favourable attitudes towards peer substance use and personal use among students at intervention universities compared to students at delayed intervention control universities.

## Conclusion

The provision of evidence-based interventions for the prevention and/or reduction of substance use to young adults is currently very limited in Germany [31]. The majority of interventions targeting young adults, including university students, are campaigns providing information on the risks and harmful consequences of substance use. Thus far, ‘social norms’-interventions have neither been implemented at multiple German universities nor have they been scientifically evaluated. Results of the INSIST-study will provide new information on the efficacy of this intervention approach in the German university context.

### Trial registration

The trial registration number of the INSIST-study is DRKS00007635 on the ‘German Clinical Trials Register’ (Date of registration: December 17^th^, 2014).
